# Exploring the link between cardiovascular risk factors and manifestations in latent tuberculosis infection: a comprehensive literature review

**DOI:** 10.1186/s43044-023-00370-5

**Published:** 2023-05-30

**Authors:** Irawaty Djaharuddin, Muzakkir Amir, Andriany Qanitha

**Affiliations:** 1 Department of Pulmonology and Respirology Medicine, Faculty of Medicine, Universitas Hasanuddin, Makassar, 90245 Indonesia; 2 Department of Cardiology and Vascular Medicine, Faculty of Medicine, Universitas Hasanuddin, Jl. Perintis Kemerdekaan Km. 10, Makassar, 90245 South Sulawesi Indonesia; 3 Department of Physiology, Faculty of Medicine, Universitas Hasanuddin, Makassar, 90245 Indonesia; 4 Doctoral Study Program, Faculty of Medicine, Universitas Hasanuddin, Makassar, 90245 Indonesia

**Keywords:** Cardiovascular disease, Myocardial infarction, LTBI, Tuberculosis

## Abstract

**Background:**

The global burden of tuberculosis (TB) and cardiovascular disease (CVD) is overt, and the prevalence of this double burden disease remains steadily rising, particularly in low- and middle-income countries. This review aims to explore the association between latent tuberculosis infection (LTBI) and the development of cardiovascular diseases and risk factors. Furthermore, we elucidated the underlying pathophysiological mechanisms that contribute to this relationship.

**Main body:**

Approximately 25% of the global population carries a dormant form of tuberculosis (TB) infection. During this latent stage, certain subsets of mycobacteria actively reproduce, and recent research suggests that latent TB infection (LTBI) is connected to persistent, long-term low-grade inflammation that can potentially contribute to the development of atherosclerosis and cardiovascular disease (CVD). The presence of LTBI can be confirmed through a positive result on either a tuberculin skin test (TST) or an interferon-gamma release assay (IGRA). Several plausible explanations for the association between LTBI and CVD include increased inflammation, autoimmunity related to heat shock proteins (HSP), and the presence of pathogens within the developing atherosclerotic plaque. The most commonly observed cardiovascular events and risk factors associated with LTBI are acute myocardial infarction, coronary artery stenosis, diabetes mellitus, and hypertension.

**Conclusions:**

This article highlights the critical role of LTBI in perpetuating the tuberculosis disease cycle and its association with cardiovascular risk factors. Chronic and persistent low inflammation underlined the association. Identifying high-risk LTBI patients and providing targeted preventive medication are crucial strategies for global TB eradication and interrupting transmission chains.

## Background

Latent tuberculosis (LTBI) affects around one-fourth of the world's population, and 10 million individuals develop TB every year [1, 2]. The two main infectious and noninfectious causes of mortality worldwide are tuberculosis and cardiovascular disease (CVD), respectively [3]. Recent research has shown that people with a history of TB have an increased risk of acute coronary syndrome [4, 5], myocardial infarction [6], ischemic strokes [7], and peripheral arterial disease [5], demonstrating that the effects of these two diseases are interrelated. Additionally, long-term CVD mortality is more likely to occur in TB patients [8, 9].

The recognition of latent tuberculosis infection (LTBI) as a condition with various host–pathogen interactions, including the possibility of intermittent mycobacterial replication and dynamic immune responses, is growing [10–12]. Previous research has demonstrated that individuals with LTBI exhibit higher levels of immune activation markers compared to those without LTBI [13, 14]. The increased immune activation in individuals with LTBI may elevate their susceptibility to developing atherosclerotic cardiovascular disease (CVD), as immune activation has been shown to contribute to atherosclerosis development [15]. In this review, our objective was to provide an updated understanding of the association between LTBI and cardiovascular risk factors and manifestations. Furthermore, we aimed to comprehensively elucidate the underlying pathomechanism that connects these two conditions.

## Main text

### Literature searching

We conducted a literature search, using the PubMed (Medline) database. We combined both MeSH and free words terms for identifying relevant articles. We also screened reference lists of published reviews to identify additional relevant studies. Details on the search strategy are presented in Table 1.

**Table 1:** Literature searching on LTBI and cardiovascular manifestations

Search	Query	Results
#5	Search: #1 OR #2 OR #3 OR #4 NOT animal	31
#4	Search: (latent tuberculosis [Title/Abstract]) AND (coronary heart disease[Title/Abstract])	1
#3	Search: (latent tuberculosis [Title/Abstract]) AND (myocardial infarction[Title/Abstract])	6
#2	Search: (latent tuberculosis [Title/Abstract]) AND (atherosclerosis[Title/Abstract])	4
#1	Search: (latent tuberculosis [Title/Abstract]) AND (cardiovascular[Title/Abstract])	26

After screening and selection of the full papers, we reached 7 articles that were eligibly included in this literature review (Table 2).

**Table 2:** A summary of the literature described the association between LTBI and cardiovascular events and risk factors

Author, year, country	Study type	Population	Settings and period of study	%Male, mean/median age	Methods to diagnose LTBI	Outcomes	Main findings
Huaman, 2018, USA[28]	Case–control	105 AMI cases vs.110 non-AMI controls	Data from 2 large national public hospital networks in Lima, Peru, between July 2015 and March 2017	69%, median age 62 (IQR 56–70 years)	The QuantiFERON-TB Gold In-Tube assay	Acute Myocardial Infarction	﻿LTBI was more frequent in AMI case patients than in controls (64% vs 49% [P = .03]; OR, 1.86; 95% CI 1.08–3.22). After adjustment LTBI remained independently associated with AMI (adjusted OR, 1.90; 95% CI, 1.05–3.45)
Huaman, 2021, USA[31]	Cross sectional	Individuals ≥ 40 years, 113 LTBI vs. 91 non-LTBI	Data from studies conducted in Lima, Peru, and Kampala, Uganda between March 2018 and October 2019	39.7%, median age 56 (IQR 49–64) years	The QuantiFERON-TB Gold Plus (QFT-Plus) was used at the Peru site. The QuantiFERON-TB Gold In-Tube (QFT-GIT) was used at the Uganda site	Obstructive CAD (plaque causing ≥ 50% stenosis)	LTBI was associated with obstructive CAD (adjusted OR, 4.96; 95% CI, 1.05–23.44; P = .043). Quantitative QFT TB antigen minus Nil interferon-γ responses were associated with obstructive CAD (adjusted OR, 1.2; 95% CI, 1.03–1.41; P = 0.022)
Khoufi, 2021, Saudi Arabia[21]	Cross sectional	98 patients with prior ischemic heart disease: 19 LTBI vs. 79 non-LTBI	Patients recruited from the outpatient cardiovascular disorders clinic and medical records of the patients at Secondary Hospital in the period from February 2018 to January 2020	62.2%, mean age 55 ± 10.1 years	QuantiFERON-TB Gold In-Tube (QFT-GIT)	Ischemic heart disease (by coronary angiography)	In multivariable analysis, LTBI was significantly associated with coronary artery atherosclerosis (Adjusted OR 1.024, 95% CI 1.002–1.736, p = 0.003)
Hasanain, 2018, Egypt[34]	Hospital‑based, case–control study	183 patients underwent percutaneous coronary angiography (121 patients with CAS vs. 62 patients without CAS)	Data from Cardiac Catheterization Unit of the Department of Cardiology, Cardiology, and Cardiac Surgery Hospital, ﻿from February 2016 to December 2017	72.7%, mean age 62.5 ± 9.9 years	Patients with positive TST and IGRA (QuantiFERON‑TB Gold (QFT‑G) test) Cellestis Ltd, Carnegie, Australia)	Coronary artery stenosis (CAS)	In multivariate analysis, LTBI (OR 2.5, 95% CI 1.2–17.3, P = 0.018) was the predictor of CAS
Erdenebat, 2018, USA[35]	Cross sectional	684 adult refugees (age ≥ 21 years)	New refugees who received care at the DeKalb County Board of Health Refugee Clinic, Atlanta, Georgia between 1st October 2013 and 31st August 2014	55.5%, median age 33 (IQR 27.0–42.0) years	QuantiFERON-TB Gold In-Tube (QFT)	Dyslipidemia	After adjusting for confounders, LTBI was not significantly associated with elevated total cholesterol (adjusted odds ratio [adjusted OR] 1.27; 95% CI 0.89–1.82) and elevated triglycerides (adjusted OR 1.18; 95% CI 0.84–1.67)
Magee, 2022, USA[36]	Retrospective cohort	574,113 Patients without preexisting diabetes	U.S. Veterans receiving care in the Veterans Health Administration from 2000 to 2015, follow-up after LTBI testing (median 3.2 years)	84%, median age of 62 (IQR 51–71) years	Tuberculin skin test (TST) or interferon-$\gamma$ release assay (IGRA)	Diabetes Mellitus	Increased diabetes persisted after adjustment for covariates (adjusted HR 1.2 [95% CI 1.2–1.3]) compared with those without LTBI
Mandieka, 2020, USA[37]	Case–control	2679 adults aged 18 to 75 with LTBI vs. 2506 LTBI-free controls	Using the Northwestern Medicine Enterprise Data Warehouse, in a large metropolitan healthcare system, between 1 January 2000 and 1 January 2020	N/A	Positive tuberculin skin test and/or interferon-γ release assay (T Spot, QuantiFERON)	Hypertension	People with LTBI had a significantly higher risk of developing hypertension (HR 2.0, 95% CI, 1.6–2.5, P < 0.001) than controls without LTBI

### Definition and diagnosis of LTBI

Classically, LTBI was defined as detectable immune sensitization to *Mycobacterium tuberculosis* (Mtb) in the absence of symptoms such as fever, chills, night sweats, weight loss, cough, hemoptysis, or a new opacity on a chest radiograph that indicates current disease. A positive result of either a tuberculin skin test (TST) or an interferon-gamma release assay (IGRA) indicates LTBI. This measurement, however, does not address the latent foci's length and activity, which differ from person to person depending on timing and host- and pathogen-specific characteristics [16]. LTBI is identified if a host has been exposed to Mtb, and a primary infection has been established. In particular, host age, immunological state, and interaction with the index case, including infectiousness and exposure, have a substantial impact on the outcome of LTBI [16].

The two currently accepted methods for LTBI screening in Mtb-exposed individuals are TST and IGRA. However, local inflammation may also be shown radiographically or pathologically 5–7 weeks after exposure [16]. TST or IGRA findings indicate Mtb infection. Patients first undergo screening for classic symptoms and signs of disease by a thorough history and physical examination in order to determine contacts of TB cases for LTBI. A chest radiograph and sputum swab for acid-fast bacilli are then necessary if the clinical suspicion is high enough to rule out an active disease.

In areas with high TB prevalence or in high-risk populations (such as those with HIV infection), more sensitive tests (such as sputum culture on liquid mercury or nucleic acid amplification tests like the XpertMtb/RIF®) may be required to completely rule out an active disease. This is important because the person who was previously categorized as having long-term lung injury (LTBI) but later found to have a positive sputum culture should now be classed as having an asymptomatic condition, which is sometimes referred to as a "subclinical" sickness. Patients who are immunocompromised, old, or toddlers are frequently misclassified according to existing classifications and testing protocols [16].

### The natural history of TB

LTBI refers to a tuberculosis infection that remains dormant and does not progress into an active disease or show any clinical symptoms [17, 18]. The global prevalence of LTBI is currently unknown due to a lack of reliable data; however, it is estimated to affect more than 33% of the world's population [1, 19–21]. Contrary to previous beliefs, LTBI is characterized by ongoing mycobacterial replication and a sustained level of immune activation. Recent research has demonstrated that individuals with LTBI exhibit consistent activation of monocytes and lymphocytes, which is not observed in healthy individuals [10]. This persistent state of immunological activation may contribute to the development of atherosclerosis and ischemic heart disease [22].

TB is mainly a lung condition. When exposed to a single droplet with 1–3 tubercle bacilli in the size range of 2–5 m, the terminal bronchioles or alveoli get infected. Experimental findings indicate that to successfully cause infection, 10–50 infectious units must be breathed [23]. The pathologic indication of original infection in humans is a solitary small tubercle, which shows that an infection was started by a solitary infectious droplet [24]. This observation calls into question whether the onset of infection results in the activation of a defense mechanism that precludes the establishment of additional infectious foci. According to experimental Mtb infection models, there is a three-day wait after the initial exposure before bacilli proliferation starts. 19–20 days pass before the 19–20 days of replication are terminated by the forming adaptive immune response. Genetics may have an impact on protective immunity because it appears to develop fairly differently in each host [16].

The majority of TB cases develop within the first two years of infection, and Mtb exposure frequently results in LTBI, which has a lifetime risk of 5–10% of developing into active tuberculosis [16]. Most of the time, primary infection is accompanied by brief, ignorable minor symptoms that hesitate people to seek medical treatment. Most initial infection cases are self-limited. However, after primary TB, there can be signs of spread to common locations for delayed reactivation To maintain TB prevalence in the community at a steady state, each TB case must infect 20 contacts, resulting in a ratio of 1:20 (pulmonary TB index case: Mtb-exposed contacts) [16]. A recent analysis revealed that each TB index case transmits 3—6 contacts [16].

Evidence showed that bacterial replication occurs in LTBI. This is supported by the well-established evidence of the effectiveness of treatment that significantly reduces the progression from LTBI as an active TB disease. Firstly, isoniazid (INH), an inhibitor of cell membrane synthesis, is the most often used medication for the chemoprevention of LTBI. Cell membrane synthesis can only take place, while an organism is actively replicating [16]. Secondly, although there is minimal induction in hypoxic environments, early secreted antigenic target of 6 kDa (ESAT-6) protein and culture filtrate protein (CFP-10)—which are employed to stimulate interferon-gamma (IFN-$\gamma$) production for IGRA testing—are released during vigorous bacterial replication [25]. Lastly, conditions of acquired immune suppression including HIV infection and treatment with tumor necrosing factor$\alpha$ (TNF-$\alpha )$ inhibitors significantly raised the likelihood of TB reactivation, further showing that certain latent foci contain viable Mtb. It is still uncertain how many "latent" Mtb foci are active and how immunosuppression affects the activity. TST and IGRA may be incorrectly negative in cases of immunosuppression or active TB disease, may revert to negative following LTBI therapy, or if the initial infection happened in the distant past, thus obscuring the present definition of LTBI [16].

The normal course of illnesses may start when a susceptible host is exposed to an infectious case of pulmonary TB. The presence of acid-fast bacilli, bacillary load, and TB infectiousness has all been linked in a previous study. Compared to pulmonary TB patients with negative smears, those with positive sputum smears are more likely to develop cavitary lung lesions with caseous necrosis, which allows for extracellular reproduction and raises the bacterial burden. They also cough more forcefully and frequently, which produces more infectious aerosols, the infectious molecule that transmits Mtb [26].

The primary lung lesion is the Ghon complex, which is frequently isolated and next to larger bronchopulmonary lymph nodes. Even while lymphadenitis is frequently not seen clinically, post-mortem research revealed that caseous necrosis is more advanced in the surrounding lymph nodes than in the lung. The size, metabolic activity, and type of the infectious focus (lymph node vs. parenchymal involvement) may all affect how quickly an LTBI develops into an active TB. Patients with positive smears for cavitary pulmonary TB vary in their level of infectiousness, and some may be "super-spreaders." Superspreaders could have the highest bacterial counts as well as the worst and longest-lasting coughs. According to a prior study, TB patients' cough intensity is a reliable predictor of high transmission [27].

### Pathomechanisms between LTBI and cardiovascular risk factors and events

Recent epidemiologic research has shown that even years after TB recovery, persons who have the disease have a higher risk of acquiring CVD than those who do not. These results suggest that TB may play a role in the development of CVD. A previous study reported that after adjusting for a 10-year cardiovascular risk score, HIV status, and recruitment location, LTBI was independently associated with a higher incidence of subclinical obstructive coronary artery disease (CAD). LTBI is an independent predictor of increased risk of atherosclerotic CVD. A recent meta-analysis including studies of patients with TB with a mean follow-up of five years reported a 1.5 times higher risk of major adverse cardiac events compared to those without TB [9]. Additionally, compared to the general population, long-term all-cause mortality is almost three times greater in people treated with TB, and the majority of this death are attributable to CVD [9].

In large population-based retrospective cohort studies, tuberculosis disease has been linked to an elevated risk of the acute coronary syndrome, ischemic stroke, and peripheral artery disease. In Taiwan, people with TB disease had an adjusted risk of 1.4 times higher suffering from acute myocardial infarction (AMI) and unstable angina compared to those without TB [4]. Similar to this, other studies also showed that patients with TB had adjusted risks for ischemic stroke and peripheral artery disease of 1.5 and 3.9 times greater, respectively, than controls without TB [5, 7]. It is important to highlight that the reference population used for this research did not distinguish between those with and without LTBI. On the other hand, Huaman et al. reported that LTBI was associated with an increased risk of acute myocardial infarction, independent of potential confounders [28].

A previous study recently reported that people with a history of TB disease have an almost twofold higher risk of developing AMI than those without prior TB who were matched for propensity score. Whether LTBI and active tuberculosis triggered CVD risk equally or gradually remains poorly understood [28]. Infection may induce atherogenesis and acute cardiovascular events through different pathways [29]. Hypothesis suggested that persistent immune activation is related to intermittent low-level microbial reproduction, a plausible potential factor in the relationship between LTBI and AMI [6]. Several studies have shown that Mtb replication and metabolic activities continue during LTBI, which is consistent with this hypothesis [10, 30].

Contrary to the previous conception that LTBI is a state of dormancy for mycobacteria; currently, LTBI is recognized as a continuous spectrum of host–pathogen interactions, where replicating and metabolically quiet mycobacterial populations coexist and are restricted by variable host immune responses within each granuloma [12, 22, 28]. Study findings have also suggested that persons with LTBI may have elevated levels of immune activation markers and proinflammatory cytokines in peripheral blood [28]. A potential pathogenic role for tuberculosis in CVD apparently presented similar mechanisms to those described for other pathogens that established chronic infection and latency. This is suggested by the fact that monocyte/macrophages, lymphocytes, and cytokines involved in cell-mediated immune responses against Mtb are also major drivers of atherogenesis.

Additionally, according to previous findings, markers for immune activation and proinflammatory cytokines may be present in greater amounts in the peripheral blood of people with LTBI. For instance, LTBI was linked to higher serum levels of interleukin (IL)-1, IL-6, and IL-22 as well as tumor necrosis factor, according to research conducted in Norway [6]. In India, LTBI was linked to increased concentrations of chemoattractive mediators like CD14, CXCL3, CCL2, and CCL8 as well as monocyte/macrophage activation markers [6, 31]. In contrast to controls without LTBI, Huaman et al*.* recently found a slight rise in plasma interferon levels in people with LTBI in the USA [28, 32].

An inflammatory profile compatible with this paradigm was observed in a Canadian study of immune activation in patients with latent and active tuberculosis. The baseline circulating levels of TNF-α, IL-1, IL-4, IL-8, and IL-22 were all considerably higher in LTBI patients compared to healthy controls with a negative tuberculin skin test (TST). In the LTBI group, IFN levels were similarly higher than in the healthy controls, while the difference was not statistically significant [6, 33].

**Fig. 1:**
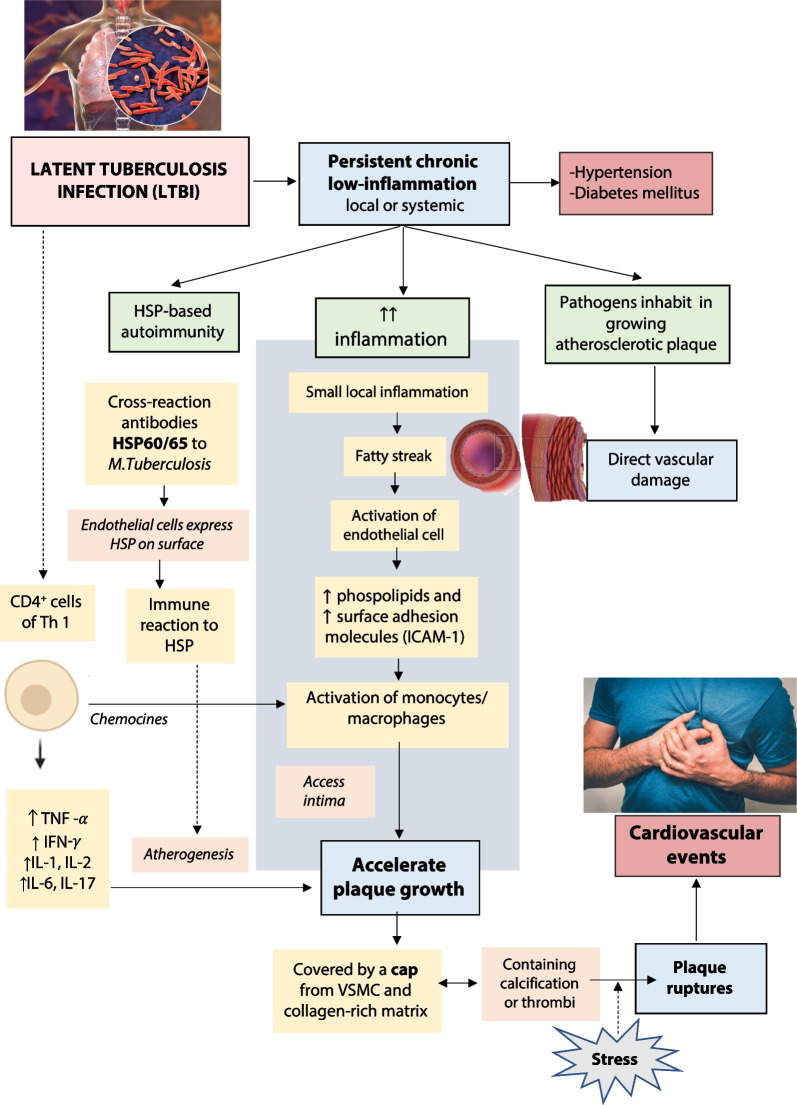
Pathomechanisms on the association between LTBI and the occurrence of cardiovascular events and risk factors

The T-cell activation markers HLA-DR and CD38 were somewhat more expressed in CD4 + and CD8 + T cells of patients with LTBI than in healthy controls, according to Wergeland et al., although the differences were not statistically significant [13]. In a study of HIV-positive patients, LTBI/HIV coinfection was found to have considerably higher CD38 expression in CD4 + and CD8 + T cells than in HIV monoinfection [14]. Even though these findings imply that immunological activation is evident in at least some categories of LTBI patients, these studies have been constrained by small sample numbers and a lack of adjustment for potential confounders. More extensive research is required to characterize immunological activation in LTBI and its possible impact on CVD. Furthermore, as LTBI treatment alters T-cell responses to particular Mtb antigens, further study of the impact of LTBI treatment on immunological activation is necessary. Detailed pathomechanisms on how LTBI related to cardiovascular disease and risk factors are described in Fig. 1.

## Conclusions

In conclusion, we shed light on the significant role of LTBI in the persistence of the disease cycle within populations, making it a crucial reservoir for new infections and ongoing transmission of Mycobacterium tuberculosis. The association between LTBI and the manifestation of cardiovascular risk factors and events, emphasizing the chronic and persistent low inflammation, observed in LTBI cases. This article contributes to the existing literature on tuberculosis and offers valuable insights for global TB eradication efforts..

The findings presented in this article support the notion that global TB eradication strategies should not solely focus on active tuberculosis cases but also consider the identification and management of LTBI. By targeting LTBI patients, public health interventions can effectively address the reservoir of Mtb within communities and prevent the development of active disease, thus breaking the cycle of transmission. Furthermore, this review underscores the need for improved diagnostic tools and risk stratification methods to accurately identify individuals with LTBI who are at the highest risk of progression. Such advancements in diagnostics and risk assessment can optimize the allocation of resources and ensure that preventive measures are implemented where they are most needed. These insights provide a foundation for future research, policy development, and implementation of effective interventions aimed at reducing the burden of tuberculosis worldwide.

## Data Availability

Not applicable.

## Abbreviations

TB: Tuberculosis
CVD: Cardiovascular disease
LTBI: Latent tuberculosis infection
Mtb: *Mycobacterium tuberculosis*
TST: Tuberculin skin test
IGRA: Interferon-gamma release assay
HIV: Human immunodeficiency virus
IFN-γ: Interferon-gamma
ESAT-6: Early secreted antigenic target of 6 kDa protein
CFP-10: Culture filtrate protein
CAD: Coronary artery disease
AMI: Acute myocardial infarction
IL: Interleukin
TNF-α: Tumor necrosing factor-alpha
